# Guidelines for superficial venous thrombosis

**DOI:** 10.1590/1677-5449.180105

**Published:** 2019-11-20

**Authors:** Marcelo José de Almeida, Ana Terezinha Guillaumon, Daniel Miquelin, Edwaldo Edner Joviliano, Ludvig Hafner, Marcone Lima Sobreira, Martin Andreas Geiger, Regina Moura, Selma Raymundo, Winston Bonnetti Yoshida

**Affiliations:** 1 Faculdade de Medicina de Marília – FAMEMA, Marília, SP, Brasil.; 2 Universidade Estadual de Campinas – UNICAMP, Campinas, SP, Brasil.; 3 Faculdade de Medicina de São José do Rio Preto – FAMERP, São José do Rio Preto, SP, Brasil.; 4 Universidade de São Paulo – USP, Faculdade de Medicina de Ribeirão Preto – FMRP, Ribeirão Preto, SP, Brasil.; 5 Universidade Estadual Paulista – UNESP, Faculdade de Medicina de Botucatu, Botucatu, SP, Brasil.

**Keywords:** clinical practice guidelines, thrombophlebitis, phlebitis, venous thrombosis, pulmonary embolism, veins

## Abstract

Superficial venous thrombosis (SVT) or superficial thrombophlebitis is characterized by thrombi within superficial veins, with partial involvement or occlusion of the lumen and inflammatory reaction along the course of the vein. Clinical diagnosis tends to be straightforward, but supplementary tests and examinations are needed to confirm thrombosis extension and possible thromboembolic complications. SVT can be associated with deep venous thrombosis in 6 to 40% of cases, with asymptomatic pulmonary embolism (PE) in 20 to 33%, and with symptomatic PE in 2 to 13%. Despite the morbidity and complications, there are currently no Brazilian guidelines for SVT. These guidelines cover the most important issues related to SVT definition, terminology, and etiology, and set out recommendations for diagnosis and treatment.

## INTRODUCTION

Superficial thrombophlebitis, or superficial venous thrombosis (SVT), is characterized by formation of thrombi inside superficial veins, with involvement or occlusion of the lumen and inflammatory reaction along the venous path. It is more common in the lower limbs and affects from 3 to 11% of the general population.^1^ Conn et al.^2^ reported prevalence of 123,000 cases/year in the United States. Clinically, SVT presents as a palpable cord and a firm area that is hot and inflamed and follows the path of a superficial vein.^3^ It can be associated with immunological syndromes (Trousseau, Lemièrre, or Mondor syndromes) or with inflammatory diseases such as thromboangiitis obliterans or thrombophilia, it can be caused by traumas or by injection of irritants, or it may be a complication of lower limb varicose veins.^3^

Clinical diagnosis tends to be straightforward, but supplementary tests and examinations are needed to confirm thrombosis extension and possible thromboembolic complications. Several types of treatments are currently available, but in general they are supported by scant scientific evidence. Existing guidelines identify options with a greater degree of consensus, some clinical and some surgical.^4^^-^ 7

Superficial venous thrombosis can be associated with deep venous thrombosis (DVT) in 6 to 40% of patients^8^^-^ 12 and can also be linked to more serious complications, such as asymptomatic pulmonary embolism (PE), in 20 to 33% of cases, and symptomatic PE, in 2 to 13% of cases.^13^^-^ 15 A recent meta-analysis^16^ reported a weighted mean prevalence of 18.2% (95% confidence interval [95%CI] 12.2-26.3%) for DVT, and 8.2% (95%CI 3.3-18.9%) for PE among patients with SVT. Rapid implementation of diagnostic and therapeutic strategies is crucial to avoiding these complications. The objective of these guidelines is to standardize treatment for SVT and report the evidence levels supporting the different treatments that are available.

## METHOD

The study organizers compiled a basic list of subjects, which was distributed to each member of the working group and a preliminary text was written. Bibliography from the previous 10 years was identified on the MEDLINE, SciELO Brasil, LILACS, Scopus, and Embase databases and publications reporting the best available evidence were selected (clinical trials, meta-analyses, and systematic reviews). Wherever possible, the PICO process (P = Patient; I = Intervention; C = Comparison; O = Outcome) was employed to formulate the search strategy.^17^ The final text was reviewed by the entire group and was agreed with full consensus between the participants. Evidence levels were classified according to the Portuguese version of the Oxford Center for Evidence-Based Medicine’s Patient Oriented Evidence That Matters definitions (Table 1).^18^

**Table 1:** Evidence levels and recommendation grades by study type, according to the Oxford Center for Evidence-Based Medicine (last updated in May 2001).^18^

**Recommendation grade**	**Evidence level**	**Treatment/** **prevention - etiology**	**Prognosis**	**Diagnosis**	**Differential diagnosis/** **prevalence of symptoms**
A	1A	Systematic review (with homogeneity) of randomized controlled clinical trials	Systematic review (with homogeneity) of cohorts since disease onset Prognostic criteria validated in different populations	Systematic review (with homogeneity) of level 1 diagnostic studies Diagnostic criteria of level 1B studies conducted at different clinical centers	Systematic review (with homogeneity) of cohort study (contemporary or prospective)
	1B	Randomized controlled clinical trials with narrow confidence interval	Cohort, since disease onset, with losses < 20% Prognostic criteria validated in a single population	Validity cohort, with good reference standard Diagnostic criteria tested at a single clinical center	Cohort study (contemporary or prospective) with few losses
	1C	“All or none” treatment results	“All or none” case-series	Sensitivity and specificity close to 100%	“All or none” case-series
B	2A	Systematic review (with homogeneity) of cohort studies	Systematic review (with homogeneity) of historical cohorts (retrospective) or of follow-up of untreated control patients in a randomized clinical trial	Systematic review (with homogeneity) of level >2 diagnostic studies	Systematic review (with homogeneity) of level ≥ 2B differential diagnosis studies
	2B	Cohort study (including randomized clinical trial of lower quality)	Historical cohort study Follow-up of untreated control patients in a randomized clinical trial Prognostic criteria derived from or validated on split samples only	Exploratory cohort study with good reference standard Diagnostic criteria derived from or validated on split samples or database	Historical cohort study (retrospective cohort) or with poor follow-up of cases (large number of losses)
	2C	Observation of treatment results (outcomes research) Ecological Study	Observation of clinical progression (outcomes research)		Ecological study
	3A	Systematic review (with homogeneity) case-control studies		Systematic review (with homogeneity) of level ≥ 3B diagnostic studies	Systematic review (with homogeneity) of level ≥ 3B studies
	3B	Case-control study		Non-consecutive case selection, or inconsistently applied reference standard	Cohort with non-consecutive case selection, or very limited study population
C	4	Case reports (including poor quality cohort or case-control studies)	Case-series (and poor quality prognostic cohort studies)	Case-control study or poor or non-independent reference standard	Case-series, or superseded reference standard
D	5	Expert opinion without critical appraisal or based on basic materials (physiological study or study with animals)			

### Questions

#### Question 1 – What is the most appropriate terminology to refer to the disease: superficial thrombophlebitis or superficial venous thrombosis of the extremities?

Traditionally, the disease has been called phlebitis or superficial thrombophlebitis. However, some authors consider that superficial venous thrombosis is a more appropriate term, because inflammation and infection are not part of the primary disease. This term is also more likely to avoid incorrect administration of antibiotics and the misconception that this is a benign disease.^4^^,^ 19 We therefore recommend using the term “superficial venous thrombosis” for this disease (Evidence level 5).

#### Question 2 – What are the etiologies of SVT of the extremities?

Etiopathogenesis of SVT is related to Virchow’s triad. The most common cause is varicose veins, because of their dilatation and tortuosity, which predisposes to stasis, inflammation, and thrombosis. Superficial venous thrombosis can also occur in patients who do not have varicosities, but have malignant diseases or diseases associated with thrombophilia, or in people who take estrogens, although the evidence for the last of these is not very well defined.^20^ Some authors classify SVT into two subclasses: related or unrelated to varicose veins.^4^ In approximately 60 to 70% of cases, SVT involves the great saphenous vein and when this is not varicose, involvement may be associated with cancer, in 5 to 13% of cases, or thrombophilia, in more than 50% of cases.^7^^,^ 21 ^-^ 23

It is also common for SVT to occur after damage to the intima caused by intravenous injection or infusion of solutions for therapeutic or diagnostic purposes, or even after mechanical injuries such as those that can occur during catheterization and hemodynamic procedures. Nowadays, many endovascular procedures are used for therapeutic purposes, primarily in the saphenous veins, to treat varicose veins, and SVT is one of the possible complications of laser, radio frequency, and even some sclerosants. Drugs, such as certain chemotherapy agents or hypertonic glucose, can often cause SVT.^24^

Certain conditions, including Buerger’s disease and syndromes such as Trousseau, Lemièrre, and Mondor, can also progress to SVT.^25^^-^ 28 Trousseau syndrome is characterized by recurrent superficial migratory thrombophlebitis and generally affects the upper and lower limbs. This syndrome is associated with malignant neoplasms and hypercoagulability, which are common in gliomas, mucin-producing adenocarcinomas of the gastrointestinal tract (stomach, pancreas, and colon), and also of the lungs, breasts, ovaries, and prostate.^20^ Lemièrre syndrome was described in 1936 and is secondary to infection, frequently of the oropharynx, that compromises the internal jugular vein, constituting septic SVT that can develop septic pulmonary emboli. These infections can be related to fitting of central catheters or even to other infections in the cervical region, usually caused by *Fusobacterium necrophorum,* a gram negative anaerobic bacteria.^29^^,^ 30 Mondor’s disease is a rare condition that is more common in females, affecting the superficial thoracic veins in the anteroposterior region. The etiology of this syndrome is unknown, but it may be related to local traumas, use of oral contraceptives, protein C deficiency, and presence of anti-cardiolipin antibodies. Some cases are also related to breast cancer.^15^^,^ 31 Another disease in which SVT can occur is thromboangiitis obliterans, also known as Buerger disease, with characteristic clinical status of migratory thrombophlebitis, which may or may not precede arterial compromise or could be concomitant.^32^

It is therefore clear that the etiology of SVT is multifactorial, in general related to Virchow’s triad (Evidence level 5). Inflammatory, chemical, biological, and infectious processes, mechanical traumas, and varicose veins are the main causes (Evidence level 5). Since varicose disease is the most frequent of these causes, SVT can be subdivided into two main groups: cases related to varicose veins and other cases (Evidence level 5).

#### Question 3 – When should thrombophilia be investigated in SVT?

Consensus statements suggest that tests for thrombophilias should not be ordered for all patients with SVT,^5^^,^ 22 even though genetic thrombophilias are an important element in predisposition for SVT, in extension of the process from the superficial system to the deep system, and also in recurrence.^28^^,^ 33 ^,^ 34 Thrombophilias should only be investigated in patients with unexplained SVT in non-varicose veins (after ruling out occult tumors) and/or those in whom thrombosis continues to progress despite the appropriate anticoagulation.^22^ Many authors consider that testing for thrombophilia in non-selected patients with DVT has no clinical value. In the 2010 British Society for Haematology consensus,^35^ recommendations were summarized as: a) who should be tested; b) who should not be tested; and c) people for whom no valid recommendation can be made with regard to the benefits of thrombophilia testing, because of a lack of evidence.

Many recommendations and suggestions are weak, because in many clinical scenarios there is only low or moderate quality evidence. Superficial venous thrombosis is related to a first manifestation of venous thrombosis in 11 to 15% of patients with protein C or S deficiency and approximately 40% of people with the F5R506Q mutation.^28^^,^ 33 ^,^ 34 ^,^ 36 ^,^ 37 However, there are no data to suggest that thrombophilia changes rates of SVT recurrence or progression.

Therefore, routinely testing patients with SVT for thrombophilia is not recommended, and the criteria in existing guidelines can be adhered to^38^ (Evidence level 1B). Several different studies report an association between SVT and hypercoagulable states, but screening is primarily recommended for patients with spontaneous SVT involving the saphenous trunks.^39^ When SVT develops in the presence of varicose veins, screening is considered unnecessary, because the SVT can be attributed to the varicose veins.^40^^,^ 41 Screening should be considered for patients with recurrent SVT after taking patient history and performing a physical examination to detect signs and symptoms consistent with cancer or other thromboembolic conditions^3^^,^ 15 (Evidence level 1B). During initial assessment of these patients, great care should be taken to investigate the possibility of personal or family history of venous thromboembolism (VTE).^42^ Laboratory tests for hereditary thrombophilia should be ordered, depending on the results of the initial patient assessment and the clinical management approach being considered^35^^,^ 43; i.e. testing is not indicated for all patients with VTE^35^^,^ 44 ^,^ 45 (Evidence level 1B).

General situations in which thrombophilia should be investigated include:

1. Unexplained SVT in non-varicose veins (after ruling out occult cancer);
2. Progression of thrombosis despite adequate anticoagulation^4^^,^ 22 ^,^ 28;
3. VTE in people younger than 40-45 years;
4. Recurrent DVT or SVT;
5. Thrombosis in unusual sites (mesenteric veins, cerebral sinus);
6. Unexplained neonatal thrombosis;
7. Skin necroses, primarily when taking coumarin;
8. Arterial thrombosis before 30 years of age;
9. Relatives of patients with prothrombotic abnormalities;
10. Patients with a clear family history of DVT;
11. Unexplained prolonged activated partial thromboplastin time (suggestive of lupus anticoagulant);
12. Recurrent pregnancy loss, immune thrombocytopenic purpura, or systemic lupus erythematosus.

#### Question 4 – Is there a concomitant relationship or correlation between SVT and VTE, and what are the risk factors?

Superficial venous thrombosis is a clinical condition that may be associated with VTE events, such as DVT and PE.^3^ Di Minno et al. conducted a meta-analysis of 4,358 patients and found that the prevalence of DVT in association with SVT was 18.1% of cases, and when prospective studies were analyzed the mean was 24%. In contrast, PE was identified in 6.9% of the patients with SVT^16^ (Evidence level 1A). In other studies, the association between DVT and PE at the time of diagnosis of SVT varies from 15 to 24.9%.^15^^,^ 23 ^,^ 25 Patients with DVT and/or PE had a 10% prevalence of SVT^46^ (Evidence level 1B).

Thrombotic involvement of the great saphenous vein, particularly close to the saphenofemoral junction in cases with varicose veins, has been identified by some authors as a risk factor for DVT and PE^15^^,^ 41 ^,^ 46 (Level 1B). Some authors consider that SVT in the great saphenous vein 3 cm from the saphenofemoral junction involves a risk of PE similar to that of DVT, and in these cases patients should be put on anticoagulation^47^^,^ 48 (Evidence level 2B). With regard to the proximity of the thrombus to the deep vein system, Galanaud et al.^49^^,^ 50 are of the opinion that patients with thrombi involving the arches or saphenofemoral/saphenopopliteal junctions should be anticoagulated, because of the increased risk of DVT. However, the presence of varicose veins does not increase the risk of VTE, although it is related to an increased recurrence of SVT. It is therefore concluded that SVT with saphenofemoral junction or saphenopopliteal junction involvement is associated with an increased risk of recurrent VTE (Evidence level 2B).

#### Question 5 – When should imaging studies be used?

##### Ultrasound

There are no studies specifically comparing the accuracy and effectiveness of different diagnostic methods for SVT. Duplex Scan (DS) has become the examination method of choice because of its low cost, effectiveness for diagnosis, and low patient risk.^51^^,^ 52 Considering the high incidence of DVT combined with the risk of progression of thrombosis and of PE,^53^ it is recommended that DM is used to examine all cases of SVT in the lower limbs^52^ (Evidence level 2B).

##### Phlebography

Phlebography does not have sufficient accuracy nor an appropriate risk-benefit profile for routine use in SVT cases.^53^^,^ 54 Even for diagnosis of DVT, its invasive nature, the exposure to radiation, and the use of iodinated contrast mean that indications are restricted to exceptional cases, such as studying reflux in pelvic vessels and compression of the left common iliac vein^48^^,^ 55 (Evidence level 2B).

##### Ventilation/perfusion scintigraphy

In studies of patients with suspected SVT and concomitant respiratory symptoms, chest pain, dyspnea, signs of PE or syncope, lung ventilation/perfusion scintigraphy can be used to diagnose PE,^3^^,^ 15 offering good accuracy (Evidence level 2B).

##### Pulmonary angiotomography

Pulmonary Computer Tomography Angiography CTA is the initial imaging method of choice for stable patients with suspected PE.^1^^,^ 2 The American College of Radiology considers chest ACT to be the current diagnostic gold standard for detection of PE^3^ (Evidence level 1B).

##### Cancer screening

Recommended for patients with SVT with no association with varicose veins, with extensive saphenous vein thrombophlebitis,^55^ with or without concomitant DVT or PE, or with recurrent or idiopathic SVT^3^^,^ 48 ^,^ 55 (Evidence level 2B).

#### Question 6 – When to indicate clinical treatment and when to indicate surgical treatment?

The objectives of SVT treatment are to: a) alleviate symptoms (reduce inflammation along the path of the veins involved and neighboring tissues); b) prevent thrombosis extension along the superficial vein system and/or into the deep system; c) avoid recurrence; and d) prevent thromboembolic complications (DVT and PE). The existence of several different series in the literature, with differing methods and contradictory results has made it difficult to standardize treatment. Treatment options are also varied: ranging from topical treatments (local heat, anti-inflammatories, and elastic compression), systemic (anti-inflammatories, heparins, antivitamin K, anti-Xa), general guidance (walking, rest in the Trendelemburg position), to surgical treatment (ligature of the great saphenous and saphenectomy).^4^

One of the indications for clinical treatment is to alleviate discomfort caused by inflammation, which is common in affected patients. In situations in which an isolated SVT of an extremity (upper or lower) is caused by a puncture complication or intravenous catheters, with compromise of collateral circulation or difficulty with infusion of osmotic solutions, it is recommended that these be withdrawn^24^ (Evidence level 4C). In situations in which the superficial venous segment involved is located in a lower limb and is compromising a saphenous trunk, clinical and/or surgical treatment options should take account of the potential causal factor, proximity with the deep vein system, any concomitant thromboembolic complications, and whether the SVT involves varicose or non-varicose veins.^15^^,^ 49 ^,^ 53

A 6-month follow-up study of 562 patients with SVT in varicose veins of the lower limbs randomized patients into five treatment groups: elastic compression only, early surgery, unfractionated heparin (UFH), low molecular weight heparin (LMWH), or warfarin.^53^ Patients were excluded if they were over the age of 70, obese, had cancer, or had DVT requiring continued full anticoagulation. The authors found that extension of the thrombus was more frequent among patients treated with elastic compression or saphenous ligature (p < 0.05), while patients treated with saphenectomy and/or stripping of segments had lower incidence of thrombus extension and greater relief from symptoms (Evidence level 1B). In SVT cases compromising varicose saphenous trunks, systemic anticoagulation with UFH, LMWH, or warfarin tends to be superior to ligature and to elastic compression in terms of thrombus extension and relief from symptoms.

The impact of SVT occurrence in varicose veins or non-varicose veins should be considered. A prospective study of 788 patients with diagnoses of SVT conducted over a 15-month period by Galanaud et al.^49^ found that occurrence of SVT in non-varicose veins increased the risk of concomitant DVT (odds ratio [OR] = 1.8; 95%CI 1.1-2.7), while occurrence of SVT in varicose veins did not exhibit a significant correlation (p > 0.05) (Evidence level 1B). Sobreira et al.^15^ reported similar findings, showing that the likelihood of DVT occurrence was more than nine times greater (OR = 9.09; 95%CI 1.75-50.00) with SVT in non-varicose veins.

**Figure 1:**
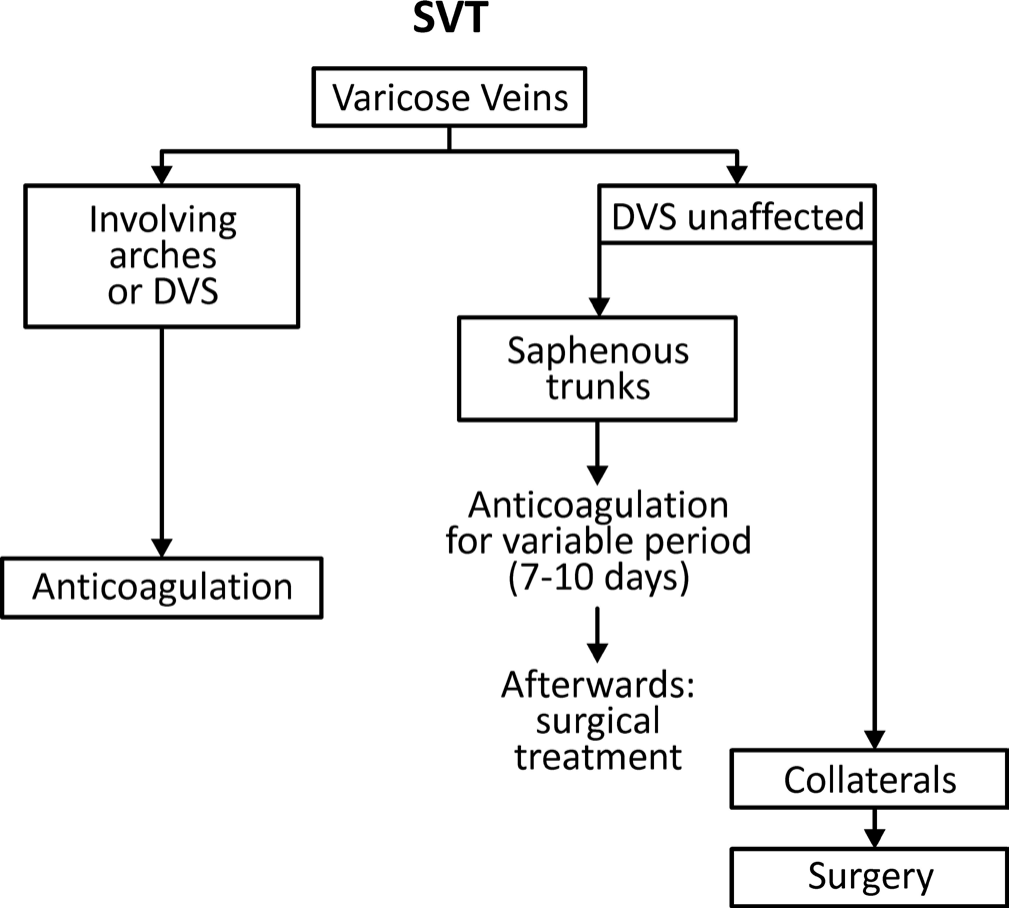
Flow diagram illustrating suggested management of treatment for SVT in varicose veins. SVT: superficial venous thrombosis; DVS: deep vein system.

**Figure 2:**
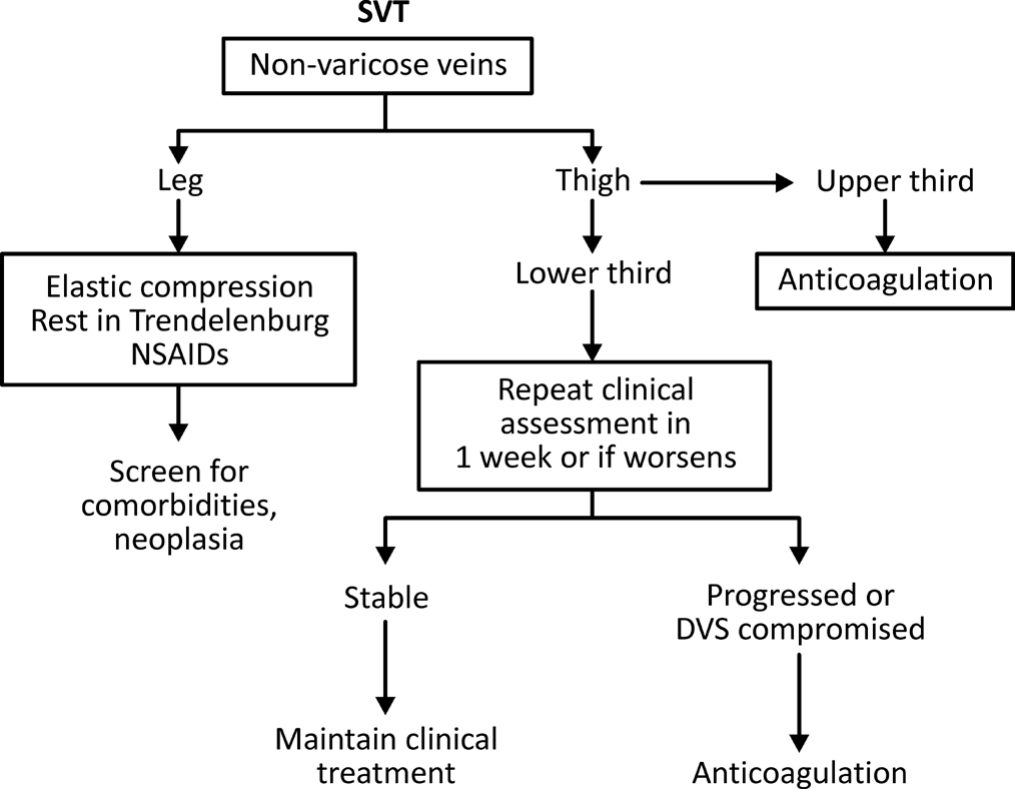
Flow diagram illustrating suggested management of treatment for SVT in non-varicose veins. SVT: superficial venous thrombosis; NSAIDs = nonsteroidal anti-inflammatories; DVS: deep vein system.

However, Gillet et al.^56^ did not detect an increase in recurrence of thromboembolic phenomena in 100 patients followed-up for up to 24 months when SVT occurred in varicose veins (16.4%), compared to when it occurred in non-varicose veins (16.7%). Systemic anticoagulation should thus be preferred in SVT cases with non-varicose saphenous trunk involvement, thereby reducing the chances of associated thromboembolic complications (Evidence level 1B) (Figures 1 and 2).

#### Question 7 – How should clinical treatment be conducted?

Clinical treatment of SVT should take account of whether collateral or tributary veins or the saphenopopliteal or saphenofemoral junctions are compromised, since this represents an increased risk of thromboembolic complications. For patients who do not have thrombus involvement in veins at the saphenous arches or increased clinical risk of DVT because of thrombophilia or associated diseases, such as cancer, treatment consists of rest, local hot compresses, use of topical agents such as heparinoids, nonsteroidal anti-inflammatories and, in combination with these treatments, graduated elastic compression stockings (GECS)^57^^-^ 59 (Evidence level 2C). Patients who do have SVT in arch veins (close to the saphenopopliteal or saphenofemoral junction), with extension exceeding 5 cm, with thrombotic risk for any reason, or with deterioration in follow-up after 7 days of clinical treatment will benefit from anticoagulants^5^^,^ 60 ^,^ 61 (Evidence level 1B).

##### Use of graduated elastic compression stockings

Use of GECS as the only treatment did not provide benefits when compared with a control group. When combined with other treatments (UFH, LMWH, nonsteroidal anti-inflammatories, and fondaparinux) they provide more accentuated clinical improvement compared to that observed in groups not using GECS.^60^ In a randomized study with 80 patients treated with LMWH and either wearing or not wearing GECS (23-32 mmHg), both groups exhibited clinical improvement and better quality of life; however, ultrasonographic findings revealed that the group that was wearing GECS had more rapid thrombus regression^62^ (Evidence level 2C).

##### Heparinoids for treatment of superficial venous thrombosis

The action of topical agents appears to provide local relief from symptoms, in addition to reducing the local inflammatory process and the intensity of pain. In previous studies, there was no difference in terms of recurrence of SVT and VTE. The patient samples in those studies were small, which is why the evidence level and recommendation grade are low^63^^,^ 64 (Evidence level 4C). Topical diclofenac proved effective in studies that also lacked more appropriate patient samples. Use of piroxican gel was not associated with differences compared to a placebo group^57^^,^ 58 (Evidence level 4C).

##### Nonsteroidal anti-inflammatories

Nonsteroidal anti-inflammatories (NSAID) reduced SVT recurrence and reduced the area affected when compared with placebo, but were inferior to heparins.^65^ According to a systematic review, exclusive use of NSAID would be indicated for reducing SVT recurrence in patients with low thromboembolic risk and in SVT with extension less than 5 cm that are not close to the saphenopopliteal or saphenofemoral junctions^5^ (Evidence level 2C).

##### Anticoagulants

Anticoagulation is indicated for patients with SVT who are at increased risk of DVT. In these cases, the frequency of VTE and the VTE recurrence rate are similar to in patients with DVT, and when present they are indications for anticoagulants, not for prolonged periods (< 3 months), although the ideal period to use these medications has not been defined. There are well-designed studies demonstrating that anticoagulants are beneficial^53^^,^ 59 ^,^ 65 ^-^ 68 (Evidence level 2B). There were no statistically significant differences in SVT regression and PE occurrence outcomes between low and high doses of nadroparin.^66^ Enoxaparin at prophylactic doses (40 mg subcutaneously[SC] once a day) was also associated with similar results for prevention of PE and reduction of SVT occurrence and extension, when compared with larger doses (1.5 mg/kg once a day).^65^ Consequently, prophylactic doses of LMWH are enough to achieve the therapeutic effect. Current recommendations are 40 mg of enoxaparin SC once a day, or 5,000 international units (IU) of dalteparin SC every 12 h, for 4 weeks (Evidence level 2A). When UFH was analyzed at doses of 5,000 IU SC twice a day and 12,500 IU SC twice a day, more favorable results were observed with the larger dosage, primarily in terms of reduction of the risk of PE, but the study had a small sample (Evidence level 2C), and so studies with larger populations are needed to better define the most appropriate dosage for this type of treatment.^67^

Fondaparinux at a dosage of 2.5mg once a day reduced symptoms and impeded SVT extension, with reduced incidence of VTE when compared to a placebo group (Evidence level 2B). In the CALISTO study,^60^ 3,002 patients were given fondaparinux (Arixtra®) 2.5 mg once a day or placebo for 45 days. Patients with SVT with 5 cm extension were included in the study; patients were excluded if they had SVT close to the saphenofemoral junction, recent surgery, prior DVT or SVT, or cancer. The results showed 0.9% complications (such as extension of SVT, DVT, or PE) in the fondaparinux group and 5.9% in the placebo group, demonstrating the efficacy of treatment with fondaparinux. However, the patients selected had low risk of complications, which could introduce bias, suggesting that it should only be used in cases with lower thromboembolic risk^5^ (Evidence level 2B).

##### Direct oral anticoagulants

Direct oral anticoagulants used to treat DVT, such as thrombin inhibitors, factor Xa inhibitors and vitamin K antagonists (VKAs), can also be used to treat SVT.^69^^-^ 72 Currently, studies are ongoing to evaluate the efficacy and cost effectiveness of these drugs for SVT-specific treatment. A meta-analysis of six studies demonstrated that using new direct oral anticoagulants was effective for prevention of TEP and recurrence of SVT, causing lower risk of bleeding compared to VKAs.^69^ The safety and efficacy of these drugs should still be assessed in further studies (Evidence level 2B). A prospective, randomized, open, multi-center non-inferiority trial compared 2.5 mg fondaparinux once a day vs. 10 mg oral rivaroxaban once a day in patients with above the knee SVT with extension greater than 5 cm (SURPRISE Trial),^70^ demonstrating similar safety and efficacy in both groups; i.e., rivaroxaban was not inferior to fondaparinux. Despite this study, rivaroxaban for SVT remains an off-label use for this medication (Evidence level 2B).

#### Question 8 – How should clinical treatment be conducted?

The longest-standing surgical treatment is ligature of the saphenous vein at the arch, with the objective of preventing propagation of the thrombus within the affected vein and into the femoral vein.^5^ This type of surgery is most indicated in cases in which there is progressive thrombus extension towards the deep vein, involving, for example, the saphenous arches.^5^ In SVT cases associated with varicose veins and with no other comorbidities, surgical treatment can avoid recurrence and reduce both symptoms and extension of the disease.^6^ Removal of the vein involved not only treats the cause of SVT, but also its complications. Another option is venous thrombectomy, primarily indicated when the SVT extends to the common femoral vein. It can also be performed in the segment of the superficial vein involved, improving symptoms more rapidly.^6^ If treated during the acute phase of SVT, the thrombus in the saphenous vein is still friable and the phleboextractor can pass easily. After a certain period, the thrombus undergoes a process of organization, preventing the surgical instrument from crossing the affected segment.

In SVT cases related to thrombophilia, with DVT in the same or contralateral limb, or in the presence of PE, surgical treatment appears to be less attractive than anticoagulants, since surgery will not directly address these complications. The conclusions of a systematic review by Di Nisio et al.^6^ pointed out that there is very limited evidence on surgical, topical, and oral treatments with relation to disease progression and emergence of thromboembolic complications. There is a lack of studies that could support a better definition of the role of surgical treatment in SVT. According to the systematic review by Di Nisio et al.,^6^ just three randomized studies were found for an analysis of surgical treatment.^53^^,^ 71 ^,^ 72

Belcaro et al.^71^ conducted an open randomized study with 83 patients with varicose veins + SVT, randomized into the following groups: A - superficial thrombectomy + GECS; B - heparin calcium + GECS; C - Venoruton® + GECS; D - thrombectomy followed by Venoruton®; or E - GECS. The outcome studied was thermography findings. Venous thrombectomy, combined or not with Venoruton®, reduced local inflammatory signs and SVT vein compromise significantly, when compared with GECS alone and with the other treatment sequences. There were no cases of DVT. While this was a randomized study, it was an open study, the number of patients in each group was too small to support more definitive conclusions, the randomization method was not described, and outcomes were limited. The level of evidence is therefore moderate or low (Evidence level 2B).

In another open multicenter randomized study conducted by Belcaro et al.,^53^ 562 patients with varicose veins + SVT were randomized to: 1 - GECS (Kendall® TED stockings); 2 - early surgery (downstream ligature or surgical removal); 3 - low dose UFH; 4 - LMWH and VKA only; or 5 - LMWH and VKA combined with late surgery. The outcomes were occurrence of DVT and SVT extension. There was no significant difference between the treatment groups in terms of DVT incidence. Surgical removal of the great saphenous vein was associated with lower SVT extension. Although the study enrolled a large number of patients (562), information on randomization and blinding of examiners to the treatments is missing, the type of surgical treatment was not defined (ligature or surgical removal), and 118 patients were lost to follow-up. For these reasons, the evidence level of this study is moderate or low (Evidence level 2B).

An open randomized study (n = 84 patients) by Lozano et al.^72^ reported that disconnection of the saphenous vein + GECS was associated with a 6.7% rate of complications, with two cases of surgical wounds, one of SVT recurrence, and two of VTE. In a group treated with LMWH + GECS for 4 weeks, the rate of complications was also 6.7%, with two cases of epistaxis, three of SVT recurrence, and no cases of VTE. The difference in the incidence of VTE was not statistically significant. The study does not provide information on the allocation process or the sample size calculation. The evidence level is moderate or low (Level 2B). As such, venous ligature with disconnection + GECS has similar results to use of LMWH + GECS in terms of complications and incidence of VTE (Evidence level 2B). Saphenectomy and venous thrombectomy may be indicated to reduce the extension and the signs and symptoms of SVT, but, apparently, without provoking any difference in the incidence of DVT or PE (Evidence level 2B)

## CONCLUSIONS

Superficial venous thrombosis is a common disease and a risk factor for thromboembolic complications similar to those related to DVT. Knowing the diagnoses and the appropriate treatment for each situation is important to ensuring patient comfort and avoiding these significant complications.
