# Supplementary material for: Guidelines for superficial venous thrombosis
Source: J Vasc Bras. 2019 Nov 20;18:e20180105. doi: 10.1590/1677-5449.180105 (PMC6880617; doi:10.1590/1677-5449.180105)
Supplement: Translation

# Translation: Diretrizes de conceito, diagnóstico e tratamento da trombose venosa superficial

**Authors:** Marcelo José de Almeida, Ana Terezinha Guillaumon, Daniel Miquelin, Edwaldo Edner Joviliano, Ludvig Hafner, Marcone Lima Sobreira, Martin Andreas Geiger, Regina Moura, Selma Raymundo, Winston Bonnetti Yoshida

**Affiliations:** 1 Faculdade de Medicina de Marília – FAMEMA, Marília, SP, Brasil.; 2 Universidade Estadual de Campinas – UNICAMP, Campinas, SP, Brasil.; 3 Faculdade de Medicina de São José do Rio Preto – FAMERP, São José do Rio Preto, SP, Brasil.; 4 Universidade de São Paulo – USP, Faculdade de Medicina de Ribeirão Preto – FMRP, Ribeirão Preto, SP, Brasil.; 5 Universidade Estadual Paulista – UNESP, Faculdade de Medicina de Botucatu, Botucatu, SP, Brasil.

**Keywords:** guia de prática clínica, tromboflebites, flebites, trombose venosa, embolia pulmonar, veias

## Abstract

A trombose venosa superficial (TVS), também denominada de tromboflebite superficial, caracteriza-se pela formação de trombos dentro de veias superficiais, com suboclusão ou oclusão da luz e reação inflamatória no seu trajeto, sendo mais comum nos membros inferiores. Afeta de 3 a 11% da população geral. O diagnóstico costuma ser clinicamente fácil, mas sua extensão e possíveis complicações tromboembólicas precisam de exames subsidiários para confirmação. A TVS pode estar associada a trombose venosa profunda em 6 a 40% dos casos, a embolia pulmonar assintomática (EP) em 20 a 33%, e a EP sintomática em 2 a 13%. Apesar de sua morbidade e das suas complicações, ainda não existem diretrizes brasileiras para a TVS. Nesta diretriz, foram analisadas as principais dúvidas quanto à definição, terminologia e etiologia da TVS, com recomendações para seu diagnóstico e tratamento.

## INTRODUÇÃO

A tromboflebite superficial, ou trombose venosa superficial (TVS), caracteriza-se pela formação de trombos dentro de veias superficiais, com suboclusão ou oclusão da luz e reação inflamatória no seu trajeto, sendo mais comum nos membros inferiores. Afeta entre de 3 a 11% da população geral^1^. Nos EUA, Conn et al.^2^ relataram uma prevalência de 123.000 casos/ano. Clinicamente, a TVS apresenta-se como cordão palpável e área endurada, quente, dolorosa e hiperemiada no trajeto de uma veia superficial^3^. Pode estar associada a síndromes imunológicas (síndromes de Trousseau, Lemièrre ou Mondor) ou a doenças inflamatórias como tromboangeíte obliterante ou trombofilia, ser causada por traumas ou injeções de substâncias irritantes, ou ainda ser uma complicação de varizes dos membros inferiores^3^.

O diagnóstico costuma ser clinicamente fácil, mas sua extensão e possíveis complicações tromboembólicas precisam de exames subsidiários para confirmação. Há vários tipos de tratamentos atualmente disponíveis, mas em geral com pouca evidência científica. Diretrizes prévias apontam algumas opções mais consensuais, tanto clínicas como cirúrgicas^4^^-^ 7.

A TVS pode estar associada a trombose venosa profunda (TVP) em 6 a 40% dos pacientes^8^^-^ 12, e também a complicações mais graves, como embolia pulmonar (EP) assintomática, em 20 a 33% dos casos, e a EP sintomática, em 2 a 13% dos casos^13^^-^ 15. Metanálise recente^16^ mostrou média ponderada de prevalência de 18,2% [intervalo de confiança de 95% (IC95%) 12,2-26,3%] de TVP, e de 8,2% (IC95% 3,3-18,9%) de EP em pacientes com TVS. A rápida adoção de estratégias diagnósticas e terapêuticas é crucial para evitar essas complicações. Esta diretriz objetiva padronizar o tratamento das TVS e demonstrar quais são os níveis de evidência dos diversos tratamentos disponíveis.

## MÉTODO

Os organizadores do estudo elaboraram uma lista de tópicos básicos, os quais foram distribuídos para cada membro do grupo, para a elaboração de um texto preliminar. Foi utilizada bibliografia dos últimos 10 anos (selecionada a partir das bases de dados MEDLINE, SciELO Brasil, LILACS, Scopus e Embase) e com a melhor evidência disponível (ensaios clínicos, metanálises e revisões sistemáticas). Sempre que cabível, foi usado o processo PICO (P = Patient; I = Intervention; C = Comparison; O = Outcome) para se constituir a estratégia de busca^17^. O texto final foi revisado pelo grupo e foi consensual entre os participantes. Os níveis de evidência foram classificados de acordo com o Oxford Centre for Evidence-Based Medicine (Centro Oxford de Medicina Baseada em Evidências) em sua versão para o português (Tabela 1)^18^.

**Tabela 1:** Níveis de evidência e graus de recomendação por tipo estudo, segundo o Oxford Centre for Evidence-Based Medicine (última atualização de maio de 2001)^18^.

**Grau de recomendação**	**Nível de evidência**	**Tratamento/** **prevenção - etiologia**	**Prognóstico**	**Diagnóstico**	**Diagnóstico diferencial/** **prevalência de sintomas**
A	1A	Revisão sistemática (com homogeneidade) de ensaios clínicos controlados e randomizados	Revisão sistemática (com homogeneidade) de coortes desde o início da doença Critérios prognósticos validados em diversas populações	Revisão sistemática (com homogeneidade) de estudos diagnósticos nível 1 Critérios diagnósticos de estudos nível 1B realizados em diferentes centros clínicos	Revisão sistemática (com homogeneidade) de estudo de coorte (contemporânea ou prospectiva)
	1B	Ensaio clínico controlado e randomizado com intervalo de confiança estreito	Coorte, desde o início da doença, com perda < 20% Critérios prognósticos validados com uma única população	Coorte validade, com bom padrão de referência Critério diagnóstico testado em um único centro clínico	Estudo de coorte (contemporânea ou prospectiva) com poucas perdas
	1C	Resultados terapêuticos do tipo “tudo ou nada”	Série de casos do tipo “tudo ou nada”	Sensibilidade e especificidade próximas de 100%	Série de casos do tipo “tudo ou nada”
B	2A	Revisão sistemática (com homogeneidade) de estudos de coorte	Revisão sistemática (com homogeneidade) de coortes históricas (retrospectivas) ou de seguimento de casos não tratados de grupo controle de ensaio clínico randomizado	Revisão sistemática (com homogeneidade) de estudos diagnósticos nível > 2	Revisão sistemática (com homogeneidade) de estudos sobre diagnóstico diferencial de nível ≥ 2B
	2B	Estudo de coorte (incluindo ensaio clínico randomizado de menor qualidade)	Estudo de coorte histórica Seguimento de pacientes não tratados de grupo controle de ensaio clínico randomizado Critério prognóstico derivado ou validado somente em amostras fragmentadas	Coorte exploratória com bom padrão de referência Critério diagnóstico derivado ou validado em amostras fragmentadas ou banco de dados	Estudo de coorte histórica (coorte retrospectiva) ou com seguimento de casos comprometido (número grande de perdas)
	2C	Observação de resultados terapêuticos (*outcomes* research) Estudo ecológico	Observação de evoluções clínicas (*outcomes* research)		Estudo ecológico
	3A	Revisão sistemática (com homogeneidade) de estudos caso-controle		Revisão sistemática (com homogeneidade) de estudos diagnósticos de nível ≥ 3B	Revisão sistemática (com homogeneidade) de estudos nível ≥ 3B
	3B	Estudo caso-controle		Seleção não consecutiva de casos, ou padrão de referência aplicado de forma pouco consistente	Coorte com seleção não consecutiva de casos, ou população de estudo muito limitada
C	4	Relato de casos (incluindo coorte ou caso-controle de menor qualidade)	Série de casos (e coorte prognóstica de menor qualidade)	Estudo caso-controle ou padrão de referência pobre ou não independente	Série de casos, ou padrão de referência superado
D	5	Opinião de especialista sem avaliação crítica ou baseada em matérias básicas (estudo fisiológico ou estudo com animais)			

### Perguntas

#### Pergunta 1 – Qual é a terminologia mais apropriada para a denominação da doença: tromboflebite superficial ou trombose venosa superficial de extremidades?

Tradicionalmente, a doença era chamada de flebite ou tromboflebite superficial. Porém, alguns autores consideraram que a denominação trombose venosa superficial seria mais adequada, porque a inflamação e a infecção não fariam parte da doença primária. Além disso, essa denominação seria mais apropriada por evitar a administração incorreta de antibióticos e também o conceito errôneo de que se trata de uma doença benigna^4^^,^ 19. Sendo assim, recomendamos a denominação “trombose venosa superficial” para essa doença (Nível de evidência 5).

#### Pergunta 2 – Quais são as etiologias da TVS de extremidades?

A etiopatogenia da TVS é relacionada à tríade de Virchow. A causa mais comum são as varizes, devido à dilatação e às tortuosidades das veias, as quais predispõem a estase, inflamação e trombose. A TVS também pode ocorrer em doentes não varicosos e que apresentam doenças malignas ou associadas à trombofilia, ou em indivíduos que utilizam estrógenos, sendo este último caso ainda sem evidência muito definida^20^. Alguns autores classificam as TVS em dois tipos: as relacionadas com varizes e as não relacionadas^4^. A TVS afeta a veia safena magna em aproximadamente 60 a 70% dos casos, e quando não é varicosa pode estar associada ao câncer, em 5 a 13% dos casos, ou à trombofilia, em mais de 50% dos casos^7^^,^ 21 ^-^ 23.

A ocorrência de TVS também é comum após lesão da íntima por substâncias químicas decorrente de injeções ou infusões intravenosas de soluções com fins terapêuticos ou diagnósticos, ou mesmo após traumas mecânicos como os que ocorrem nos procedimentos de cateterismo e hemodinâmica. Hoje em dia se realizam vários procedimentos endovasculares com finalidade terapêutica, principalmente nas veias safenas, para obter o tratamento de varizes, sendo a TVS uma das possíveis complicações do uso de laser, radiofrequência ou mesmo de alguns esclerosantes. Drogas, como alguns quimioterápicos ou glicose hipertônica, podem com frequência causar TVS^24^.

Existem também algumas enfermidades, incluindo a doença de Buerger e síndromes tais como as de Trousseau, Lemièrre e Mondor, que podem evoluir com TVS^25^^-^ 28. A síndrome de Trousseau é caracterizada por tromboflebites migratórias superficiais recorrentes e afeta comumente os membros superiores e inferiores. Essa síndrome está associada a neoplasias malignas e a hipercoagulabilidade, que são frequentes em gliomas, adenocarcinomas do trato gastrointestinal produtores de mucina (estômago, pâncreas e cólon), e também de pulmão, mama, ovário e próstata^20^. A síndrome de Lemièrre foi descrita em 1936 e é secundária a um quadro infeccioso, frequentemente da orofaringe, que compromete a veia jugular interna, caracterizando um quadro de TVS séptica que pode evoluir para êmbolos sépticos para o pulmão. Essas infecções podem estar relacionadas à colocação de cateteres centrais ou mesmo a outras infecções na região cervical usualmente causadas pelo *Fusobacterium necrophorum,* uma bactéria anaeróbia gram negativa^29^^,^ 30. A doença de Mondor é rara e mais frequente no sexo feminino, atingindo as veias superficiais torácicas na região anteroposterior. A etiologia dessa síndrome é desconhecida, mas pode estar relacionada a traumas locais, uso de anticoncepcionais orais, deficiência de proteína C e presença de anticorpos anticardiolipinas. Alguns casos se relacionam também à presença de câncer de mama^15^^,^ 31. Outra doença em que pode ocorrer a TVS é a tromboangeíte obliterante, também chamada doença de Buerger, cujo quadro clínico se caracteriza pela tromboflebite migratória, que pode preceder ou não ao comprometimento arterial ou mesmo ser concomitante^32^.

Sendo assim, a etiologia da TVS é multifatorial, em geral vinculada à tríade de Virchow (Nível de evidência 5). Processos inflamatórios, químicos, biológicos, infecciosos, traumas mecânicos e varizes constituem as suas principais causas (Nível de evidência 5). Por ser a doença varicosa sua causa mais frequente, a TVS pode dividida em dois grandes grupos: a relacionada com varizes e as demais (Nível de evidência 5).

#### Pergunta 3 – Quando investigar trombofilia na TVS?

Consensos sugerem não pesquisar trombofilia para qualquer paciente com TVS^5^^,^ 22, embora trombofilias genéticas representem um elemento importante na predisposição para TVS, na extensão do processo do sistema superficial para o profundo, e também na sua recorrência^28^^,^ 33 ^,^ 34. Essa pesquisa deve ser realizada somente em pacientes com TVS não explicada em veias não varicosas (após exclusão de uma neoplasia oculta) e/ou naqueles com progressão do trombo apesar de apropriada anticoagulação^22^. Muitos autores acreditam que a pesquisa de trombofilia em pacientes com TVP não selecionados não teria valor clínico. No consenso da The British Society for Haematology^35^ em 2010, as recomendações foram resumidas em: a) quem deveria ser testado; b) quem não deveria ser testado; e c) indivíduos para os quais nenhuma recomendação válida pode ser feita a respeito do benefício de pesquisar trombofilia, devido à falta de evidências.

Muitas das recomendações e sugestões são fracas, pois em muitos cenários clínicos há somente baixa ou moderada qualidade de evidência. A TVS está relacionada com uma primeira manifestação de trombose venosa em 11 a 15% de pacientes com deficiência de proteína C ou S e em aproximadamente 40% daqueles com a mutação F5R506Q^28^^,^ 33 ^,^ 34 ^,^ 36 ^,^ 37. Entretanto, não há dados para sugerir que a presença de trombofilia poderia alterar as taxas de recorrência ou progressão de TVS.

Sendo assim, a investigação de pacientes com TVS para trombofilia não é rotineiramente indicada, e poderia seguir critérios de diretrizes existentes^38^ (Nível de evidência 1B). Vários estudos relatam associação de TVS com estados hipercoaguláveis, mas o rastreamento é recomendado principalmente para pacientes com TVS espontânea com envolvimento dos troncos das veias safenas^39^. Quando a TVS se desenvolve na presença de veias varicosas, esse rastreamento é considerado desnecessário, pois o episódio de TVS é atribuído às veias varicosas^40^^,^ 41. O rastreamento deve ser considerado para pacientes com TVS recorrente após história e exame físico para avaliar sinais e sintomas consistentes com neoplasia ou outras condições tromboembólicas^3^^,^ 15 (Nível de evidência 1B). Na avaliação inicial desses pacientes, deve-se investigar minuciosamente na anamnese e o antecedente pessoal ou familiar de tromboemboembolismo venoso (TEV)^42^. Testes laboratoriais para detecção de trombofilia hereditária devem ser solicitados, dependendo do resultado da avaliação inicial do paciente e da abordagem clínica a ser considerada^35^^,^ 43; portanto, não é indicada sua realização em qualquer paciente com TEV^35^^,^ 44 ^,^ 45 (Nível de evidência 1B).

As situações gerais em que a trombofilia deve ser investigada incluem:

1. TVS inexplicável em veias não varicosas (após exclusão de câncer oculto);
2. Progressão do trombo apesar de anticoagulação adequada^4^^,^ 22 ^,^ 28;
3. TEV em pessoas com idade inferior a 40-45 anos;
4. TVP ou TVS recorrentes;
5. Trombose em sítios não usuais (veias mesentéricas, seio cerebral);
6. Trombose neonatal inexplicável;
7. Necroses de pele, principalmente em uso de cumarina;
8. Trombose arterial antes dos 30 anos;
9. Parentes de pacientes com anormalidade trombofílica;
10. Pacientes com história familiar clara de TVP;
11. Tempo de tromboplastina parcial ativado prolongado inexplicado (sugere anticoagulante lúpico);
12. Perda fetal recorrente, púrpura trombocitopênica imune ou lúpus eritematoso sistêmico.

#### Pergunta 4 – Existe concomitância ou correlação entre TVS e TEV, e quais são os fatores de risco?

A TVS é uma condição clínica que pode estar associada a eventos de TEV, como TVP e EP^3^. Di Minno et al., em metanálise com 4.358 pacientes, encontraram uma prevalência de TVP associada a TVS em 18,1% dos casos, e, quando foram analisados os estudos prospectivos, a média foi de 24%. Já EP foi identificada em 6,9% dos pacientes com quadro de TVS^16^ (Nível de evidência 1A). Em outros estudos, a associação entre TVP e EP, no momento do diagnóstico de TVS, variou entre 15 e 24,9%^15^^,^ 23 ^,^ 25. Pacientes com TVP e/ou EP apresentaram prevalência de 10% de TVS^46^ (Nível de evidência 1B).

O envolvimento trombótico da veia safena magna, principalmente nas proximidades da junção safenofemoral, nos casos com varizes, foi apontado por alguns autores como sendo fator de risco de TVP e EP^15^^,^ 41 ^,^ 46 (Nível 1B). Alguns autores chegaram a considerar que a TVS de veia safena magna a 3 cm da junção safenofemoral apresenta risco de EP semelhante ao da TVP, sendo que para esses casos, os pacientes devem ser submetidos a anticoagulação^47^^,^ 48 (Nível de evidência 2B). Em relação à proximidade do trombo em relação ao sistema venoso profundo, Galanaud et al.^49^^,^ 50 também opinaram que os pacientes com trombos atingindo as crossas ou a junção safenofemoral/safeno-poplítea devem ser anticoagulados, devido ao risco aumentado de TVP; entretanto, a presença de varizes não aumentou o risco de TEV, embora haja aumento na recorrência da TVS. Conclui-se, por fim, que a TVS com envolvimento da junção safenofemoral ou safeno-poplítea influenciou no aumento do risco de TEV recorrente (Nível de evidência 2B).

#### Pergunta 5 – Quando fazer estudo de imagem?

##### Ultrassom

Não existem estudos específicos comparando acurácia e eficiência entre diferentes métodos diagnósticos para TVS. O mapeamento dúplex (MD) tem sido o exame de escolha devido ao seu baixo custo, eficiência no diagnóstico e baixo risco para os pacientes^51^^,^ 52. Considerando a alta incidência de TVP associada ao risco de progressão da trombose e da EP^53^, sugere-se a realização do MD em todos os casos de TVS em membros inferiores^52^ (Nível de evidência 2B).

##### Flebografia

A flebografia não apresenta acurácia e nem risco beneficio adequados para sua utilização rotineira nos casos de TVS^53^^,^ 54. Mesmo para diagnóstico da TVP, em razão de ser exame invasivo, da exposição à radiação e do uso de contraste iodado, sua indicação tem se restringido para casos excepcionais como estudo de refluxo em vasos pélvicos e da compressão da veia ilíaca comum esquerda^48^^,^ 55 (Nível de evidência 2B).

##### Cintilografia de ventilação/perfusão

Em estudos de pacientes com suspeita de TVS com sintomas respiratórios concomitantes, dor torácica, dispneia, sinais de EP ou síncope, a cintilografia de ventilação/perfusão do pulmão pode ser usada para o diagnóstico de EP^3^^,^ 15, por apresentar boa acurácia (Nível de evidência 2B).

##### Angiotomografia pulmonar

A angiotomografia pulmonar (ANGIO-TC) é a modalidade de imagem inicial de escolha para pacientes estáveis com suspeita de EP^1^^,^ 2. O Colégio Americano de Radiologia considera que a ANGIO-TC de tórax é o padrão-ouro atual no diagnóstico para a detecção de EP^3^ (Nível de evidência 1B).

##### Rastreamento para neoplasia

Indicado em pacientes com TVS não associada a veias varicosas, tromboflebite extensa da veia safena^55^, com ou sem TVP ou EP concomitante, ou com TVS recorrente ou idiopática^3^^,^ 48 ^,^ 55 (Nível de evidência 2B).

#### Pergunta 6 – Quando indicar tratamento clínico e quando indicar o tratamento cirúrgico?

O tratamento da TVS tem como objetivo: a) promover alívio sintomático (reduzir a inflamação do trajeto venoso comprometido, assim como dos tecidos vizinhos); b) prevenir a extensão do trombo para o interior do sistema venoso superficial e/ou profundo; c) evitar recorrência; d) prevenir complicações tromboembólicas (TVP e EP). A existência de várias séries na literatura, com metodologias heterogêneas e resultados controversos, tem dificultado a padronização terapêutica. As modalidades terapêuticas também são variadas: desde tratamentos tópicos (calor local, anti-inflamatórios e compressão elástica), sistêmicos (anti-inflamatórios, heparinas, antivitamina K, anti-Xa), orientações gerais (deambulação, repouso em Trendelemburg), até o tratamento cirúrgico (ligadura de safena magna e safenectomia)^4^.

Uma das indicações do tratamento clínico é o alívio do desconforto devido ao quadro inflamatório, ocorrência comum nos pacientes afetados. Em situações em que a TVS isolada de extremidade (superior ou inferior) for decorrente de complicação de punção ou de cateteres intravenosos com comprometimento da circulação colateral ou dificuldade de infusão de soluções osmóticas, recomenda-se que estes sejam retirados^24^ (Nível de evidência 4C). Em situações em que o segmento venoso superficial comprometido está localizado na extremidade inferior e compromete um tronco safeno, as opções terapêuticas clínicas e/ou cirúrgicas devem levar em conta o potencial fator causal, a proximidade com o sistema venoso profundo, a concomitância de complicação tromboembólica, e se a TVS ocorre em veias varicosas ou em veias não varicosas^15^^,^ 49 ^,^ 53.

Em estudo de 562 pacientes com TVS em veias varicosas de membros inferiores com seguimento de 6 meses, os pacientes foram randomizados em cinco grupos de tratamento: somente a compressão elástica, cirurgia precoce, heparina não fracionada (HNF), heparina de baixo peso molecular (HBPM) e varfarina^53^. Foram excluídos pacientes com mais de 70 anos, obesos, oncológicos ou com TVP associada que necessitariam de extensão de anticoagulação plena. Os autores verificaram que a extensão do trombo ocorreu mais frequentemente nos pacientes submetidos a compressão elástica e a ligadura de safena (p < 0,05), enquanto nos pacientes submetidos a safenectomia e/ou retirada dos trajetos houve menor incidência de extensão do trombo e maior alívio sintomático (Nível de evidência 1B). Em casos de TVS comprometendo troncos safenos varicosos, a anticoagulação sistêmica com HNF, HBPM ou varfarina costuma ser superior à ligadura e à compressão elástica em termos de extensão do trombo e alívio sintomático.

Deve ser ressaltado o impacto da ocorrência da TVS em veias varicosas ou veias não varicosas. Em estudo prospectivo de 788 pacientes com diagnóstico de TVS realizado durante um período de 15 meses, Galanaud et al.^49^ verificaram que a ocorrência de TVS em veias não varicosas aumentou o risco para TVP concomitante [*odds ratio* (OR) = 1,8; IC95%1,1-2,7], enquanto a ocorrência da TVS em veias varicosas não mostrou correlação significativa (p > 0,05) (Nível de evidência 1B). Fato semelhante foi encontrado por Sobreira et al.^15^, sendo que a chance de ocorrência de TVP aumentou em mais de nove vezes (OR = 9,09; IC95% 1,75-50,00) em veias não varicosas.

Por outro lado, Gillet et al.^56^ não encontraram aumento da recorrência de fenômenos tromboembólicos em seguimento de 100 pacientes por um período de até 24 meses quando a TVS ocorria em veias varicosas (16,4%) em relação a veias não varicosas (16,7%). Desse modo, a anticoagulação sistêmica deve ser preferida em casos de TVS comprometendo troncos safenos não varicosos, diminuindo-se assim a chance de ocorrência de complicações tromboembólicas associadas (Nível de evidência 1B) (Figuras 1 e 2).

**Figura 1:** Fluxograma com sugestão de manejo de tratamento da TVS em veias varicosas. TVS: trombose venosa superficial; SVP: sistema venoso profundo.

**Figura 2:** Fluxograma com sugestão de manejo de tratamento da TVS em veias não varicosas. TVS: trombose venosa superficial; AINH = anti-inflamatórios não hormonais; SVP: sistema venoso profundo.

#### Pergunta 7 – Como fazer o tratamento clínico?

O tratamento clínico da TVS deve levar em conta se o acometimento ocorreu em veias colaterais e tributárias ou nas junções safeno-poplítea e safenofemoral, que apresentam maior risco de complicações tromboembólicas. Para pacientes que não apresentam acometimento por trombo em veias da crossa da safena e nem risco clínico aumentado de TVP por trombofilia ou doenças associadas, como neoplasias, o tratamento consiste em repouso, compressas locais quentes, uso de agentes tópicos como heparinoides, anti-inflamatórios não hormonais e, em associação a esses tratamentos, meias elásticas de compressão graduada (MECG)^57^^-^ 59 (Nível de evidência 2C). Os pacientes que apresentam TVS em veias das crossas (próximas à junção safeno-poplítea ou safenofemoral), com mais de 5 cm de extensão, com risco trombótico por qualquer causa, ou com piora no acompanhamento após 7 dias de tratamento clínico se beneficiam com uso de anticoagulantes^5^^,^ 60 ^,^ 61 (Nível de evidência 1B).

##### Uso de meias elásticas de compressão graduada

A utilização de MECG como único tratamento não apresenta benefícios quando comparada ao grupo controle. Quando associada a outros tratamentos (HNF, HBPM, anti-inflamatórios não esteroides e fondaparinux) proporciona melhora clínica mais acentuada se comparada àquela observada no grupo sem uso de MECG^60^. Em estudo randomizado com 80 pacientes tratados com HBPM e utilizando ou não MECG (23-32 mmHg), houve melhora clínica e da qualidade de vida em ambos os grupos; entretanto, achados ultrassonográficos revelaram que o grupo que utilizou MECG apresentou regressão mais rápida do trombo^62^ (Nível de evidência 2C).

##### Heparinoides no tratamento da trombose venosa superficial

A ação dos agentes tópicos parece proporcionar alívio local dos sintomas, além de levar à redução do processo inflamatório local e da intensidade da dor. Em estudos prévios, não houve diferenças quanto à recorrência da TVS e do TEV. A casuística dos estudos foi pequena, e por isso o nível de evidência e o grau de recomendação são baixos^63^^,^ 64 (Nível de evidência 4C). O uso de diclofenaco tópico demonstrou ser efetivo em estudos que também carecem de uma casuística mais apropriada. O uso do piroxican gel não apresentou diferenças quando comparado ao grupo placebo^57^^,^ 58 (Nível de evidência 4C).

##### Anti-inflamatórios não hormonais

Os anti-inflamatórios não hormonais (AINH) promoveram redução da recorrência da TVS e da área acometida quando comparados ao placebo; porém, foram inferiores quando comparados com as heparinas^65^. Em revisão sistemática, o uso exclusivo de AINH estaria indicado para redução da recorrência de TVS em pacientes com baixo risco tromboembólico e em TVS com menos de 5 cm de extensão e não próxima à região das junções safeno-poplítea e safenofemoral^5^ (Nível de evidência 2C).

##### Anticoagulantes

A anticoagulação é indicada para pacientes com TVS que apresentam risco aumentado de TVP. Nesses casos, a frequência do TEV ou sua recorrência são similares às dos pacientes com TVP, e quando presentes indicam necessidade de uso de anticoagulantes por períodos não estendidos (< 3 meses); não há definição sobre qual o tempo ideal de uso dessas medicações. Há estudos bem delineados que demonstram o benefício do uso de anticoagulantes^53^^,^ 59 ^,^ 65 ^-^ 68 (Nível de evidência 2B). Quanto ao uso de nadroparina, não houve diferenças estatisticamente significativas entre baixas ou altas doses^66^ quando observados resultados de regressão da TVS e ocorrência de EP. O uso da enoxaparina em doses profiláticas – 40 mg por via subcutânea (SC) uma vez ao dia –também apresentou resultados semelhantes na prevenção de EP e na diminuição da recorrência e extensão da TVS, quando comparado com doses maiores (1,5 mg/kg uma vez ao dia)^65^. Consequentemente, doses profiláticas de HBPM são suficientes para o efeito terapêutico. Indica-se atualmente enoxaparina 40 mg SC, uma vez ao dia ou dalteparina 5.000 unidades internacionais (UI) SC, de 12 em 12 h, por 4 semanas (Nível de evidência 2A). Quando se analisou a HNF nas doses de 5.000 UI SC duas vezes ao dia e 12.500 UI SC duas vezes ao dia, houve resultados mais favoráveis com o uso de uma dose maior, principalmente na redução do risco de EP; entretanto, o estudo envolveu uma casuística pequena (Nível de evidência 2C), havendo portanto necessidade de estudos com casuísticas maiores para melhor definir a dosagem mais adequada para esse tipo de tratamento^67^.

O fondaparinux na dose de 2,5mg uma vez ao dia reduziu os sintomas e impediu o aumento da extensão da TVS, com diminuição da incidência da TEV quando comparado ao grupo placebo (Nível de evidência 2B). No estudo CALISTO^60^, 3.002 pacientes receberam fondaparinux (Arixtra®) 2,5 mg uma vez ao dia ou placebo por 45 dias. Pacientes com TVS com 5 cm de extensão foram incluídos no estudo; foram excluídos pacientes com TVS próxima à junção safenofemoral, cirurgia recente, TVP ou TVS prévia e câncer. Os resultados mostraram apontaram 0,9% de complicações (como extensão da TVS, TVP e EP) no grupo fondaparinux, e 5,9% no grupo placebo, demonstrando a eficácia do tratamento com fondaparinux. Entretanto, foram selecionados pacientes com baixo risco de complicações, o que pode ser um viés, sugerindo seu uso apenas para casos com risco tromboembólico mais reduzido^5^ (Nível de evidência 2B).

##### Anticoagulantes orais diretos

Os anticoagulantes orais diretos utilizados no tratamento da TVP, como os inibidores da trombina e de anti Xa e os antagonistas da vitamina K (AVKs), também podem ser usados no tratamento da TVS^69^^-^ 72. Atualmente, estudos estão sendo realizados para avaliar a eficácia e o custo efetividade dessas drogas no tratamento específico da TVS. Uma metanálise de seis estudos demonstrou que o uso de novos anticoagulantes orais diretos foi efetivo na prevenção do TEP e na recorrência da TVS, causando menor risco de sangramentos quando comparado ao uso de AVKs^69^. A segurança e a eficácia dessas drogas deverão ainda ser avaliadas em mais estudos (Nível de evidência 2B). Em estudo randomizado, prospectivo, de não inferioridade, aberto e multicêntrico comparando fondaparinux 2,5 mg uma vez ao dia *versus* rivaroxabana 10 mg via oral uma vez ao dia em pacientes com TVS supragenicular com mais de 5 cm de extensão (SURPRISE Trial)^70^, demonstrou-se segurança e eficácia semelhantes nos dois grupos; portanto, rivaroxabana foi não inferior ao fondaparinux. Apesar desse estudo, o uso de rivaroxabana para TVS ainda não está indicado na bula do medicamento (Nível de evidência 2B).

#### Pergunta 8 – Como fazer o tratamento cirúrgico?

O tratamento cirúrgico mais antigo é a ligadura da veia safena junto à sua crossa, com o objetivo de evitar a propagação do trombo na veia acometida e para dentro da veia femoral^5^. Esse tipo de cirurgia é mais indicado nos casos em que há progressiva extensão do trombo para veia profunda, envolvendo, por exemplo, a crossa das veias safenas^5^. Nos casos de TVS associada a varizes e sem outras comorbidades, o tratamento cirúrgico pode evitar recorrências e reduzir os sintomas e a extensão da doença^6^. A retirada da veia acometida trata não só a causa mas também as complicações da TVS. Além disso, a trombectomia venosa é uma alternativa indicada principalmente quando há extensão da TVS para a veia femoral comum. Pode ser realizada também no segmento da veia superficial acometida, melhorando a sintomatologia mais rapidamente^6^. Ainda na fase aguda da TVS, o trombo presente na veia safena ainda é friável e permite a passagem fácil do fleboextrator. Depois de algum tempo, o trombo sofre um processo de organização, o que impossibilita a passagem desse instrumento cirúrgico pelo segmento acometido.

Nos casos de TVS relacionadas com trombofilia, de TVP associada no mesmo membro ou no membro contralateral, ou mesmo de EP, o tratamento cirúrgico parece ser menos interessante que o uso de anticoagulantes, uma vez que a cirurgia não trataria diretamente essas complicações. A conclusão da revisão sistemática de Di Nisio et al.^6^ apontou que os tratamentos cirúrgico, tópico e oral têm evidências muito limitadas a respeito da progressão da doença e do aparecimento de complicações tromboembólicas. Faltam estudos para se definir melhor o papel do tratamento cirúrgico na TVS. De acordo com revisão sistemática de Di Nisio et al.^6^, apenas três estudos randomizados foram encontrados para análise do tratamento cirúrgico^53^^,^ 71 ^,^ 72.

No estudo randomizado aberto de Belcaro et al.^71^, 83 pacientes com varizes + TVS foram randomizados nos seguintes grupos: A - trombectomia superficial + MECG; B - heparina cálcica + MECG; C - Venoruton® + MECG; D - trombectomia seguida de Venoruton®; E - MECG. O desfecho estudado foram as alterações da termografia. A trombectomia venosa, associada ou não ao Venoruton®, reduziu significativamente os sinais locais inflamatórios e as veias comprometidas por TVS, comparada com MECG isolada e com os demais esquemas de tratamento. Nenhum caso teve TVP. Embora seja um estudo randomizado, foi um estudo aberto, o número de pacientes por grupo foi pequeno para fornecer conclusões mais definitivas, o método de aleatorização não foi descrito, e os desfechos foram limitados. Portanto, o grau de evidência é moderado ou baixo (Nível de evidência 2B).

Em outro estudo multicêntrico randomizado aberto realizado por Belcaro et al.^53^, 562 pacientes com varizes + TVS foram randomizados para: 1 - MECG (meias TED Kendall®); 2 - cirurgia precoce (ligadura a jusante ou remoção cirúrgica); 3 - HNF baixas doses; 4 - HBPM e AVK isolados; 5 - HBPM e AVK associados a cirurgias tardias. Os desfechos foram ocorrência de TVP e extensão da TVS. Não houve diferença significante entre os grupos de tratamento quanto à incidência de TVP. A remoção cirúrgica da veia safena magna foi associada com menor extensão da TVS. Apesar de o estudo incluir um número grande de pacientes (562), há falta de informações sobre aleatorização e sobre o cegamento dos avaliadores quanto aos tratamentos efetuados, não foi discriminado o tipo de tratamento cirúrgico (ligadura ou retirada cirúrgica), e houve perda de 118 pacientes. Por esses motivos, o grau de evidência desse estudo é moderado ou baixo (Nível de evidência 2B).

No estudo randomizado aberto (n = 84 pacientes) de Lozano et al.^72^, a desconexão da veia safena + MECG apresentou uma taxa de complicações de 6,7%, sendo dois casos da ferida operatória, um de recorrência da TVS e dois de TEV. No grupo tratado com HBPM + MECG por 4 semanas, houve também 6,7% de complicações, sendo dois casos de epistaxe, três de recorrência de TVS e nenhum de TEV. A diferença de incidência de TEV não foi estatisticamente significante. Nesse estudo, não houve informação sobre o processo de alocação e o cálculo de tamanho de amostra. O grau de evidência é moderado ou baixo (Nível 2B). Sendo assim, a ligadura venosa com desconexão + MECG tem resultado similar ao do uso de HBPM + MECG em termos de complicações e incidência de TEV (Nível de evidência 2B). A safenectomia e a trombectomia venosa poderiam ser indicadas para reduzir a extensão e os sinais e sintomas da TVS, mas aparentemente, sem promover diferença quanto à incidência de TVP e de EP (Nível de evidência 2B)

## CONCLUSÃO

A TVS é uma doença frequente e representa fator de risco para complicações tromboembólicas similares às da TVP. O conhecimento do diagnóstico e do tratamento adequados em cada situação é importante no sentido de trazer conforto ao paciente e de evitar essas complicações importantes.
